# Cell Transformation by PTP1B Truncated Mutants Found in Human Colon and Thyroid Tumors

**DOI:** 10.1371/journal.pone.0166538

**Published:** 2016-11-17

**Authors:** Wenhan Mei, Kemin Wang, Jian Huang, Xinmin Zheng

**Affiliations:** 1 Department of Biochemistry and Molecular Cell Biology, Shanghai Key Laboratory of Tumor Microenvironment and Inflammation, Shanghai Jiao Tong University School of Medicine (SJTU-SM), Shanghai, 200025, China; 2 Department of Molecular Biology and Genetics, Cornell University, Ithaca, NY, United States of America; Rush University Medical Center, UNITED STATES

## Abstract

Expression of wild-type protein tyrosine phosphatase (PTP) 1B may act either as a tumor suppressor by dysregulation of protein tyrosine kinases or a tumor promoter through Src dephosphorylation at Y527 in human breast cancer cells. To explore whether mutated PTP1B is involved in human carcinogenesis, we have sequenced PTP1B cDNAs from human tumors and found splice mutations in ~20% of colon and thyroid tumors. The PTP1BΔE6 mutant expressed in these two tumor types and another PTP1BΔE5 mutant expressed in colon tumor were studied in more detail. Although PTP1BΔE6 revealed no phosphatase activity compared with wild-type PTP1B and the PTP1BΔE5 mutant, its expression induced oncogenic transformation of rat fibroblasts without Src activation, indicating that it involved signaling pathways independent of Src. The transformed cells were tumourigenic in nude mice, suggesting that the PTP1BΔE6 affected other molecule(s) in the human tumors. These observations may provide a novel therapeutic target for colon and thyroid cancer.

## Introduction

It is widely accepted that cancer has a genetic basis and that mutations affecting the coding sequences of specific genes are hallmarks of cancer. The identification of the genetic alterations associated with a human cancer has a dramatic impact on every aspect of the field from understanding basic mechanisms of carcinogenesis to diagnosis and treatment. Protein tyrosine phosphorylation plays a crucial role in many cellular signal transduction pathways, including those affecting growth, differentiation, cell cycle regulation, apoptosis, and invasion[1]. This reversible phosphorylation is coordinately controlled by protein tyrosine kinases (PTKs) and phosphatases (PTPs) that keep a dynamic equilibrium[2]. Mounting evidence supports the concept that carcinogenesis can be partly ascribed to dysregulation between PTKs and PTPs[3,4]. In addition, a variety of PTK and PTP genes have been directly linked to tumorigenesis through somatic mutations[5].

The PTPs represent a large superfamily that composed of 107 members[6]. Aberrant PTPs have been found in many diseases including malignant tumors. In our previous reports, we defined the molecular mechanism for the activation of PTPα and regulation of its Src-mediated transforming activity [7–9].

The protein tyrosine phosphatase 1B (PTP1B) is a classical non-receptor protein tyrosine phosphatase that is an important regulator of signaling pathways involved in human diseases such as obesity, diabetes, and cancer. The human PTP1B gene is comprised of 10 exons. PTP1B protein consists of 435 amino acids and features an N-terminal catalytic domain, two proline-rich motifs, and a C-terminal hydrophobic region [10].

The highly conserved N terminal domain of PTP1B, also known as the P-loop, is the active center encoded by exon6, composed of amino acid residues 214–221 (HCSAGTGR).Cys215 is the key catalytic site. The proline-rich WpD loop, which contains Asp181 and Phe182, is responsible for the recognition of the substrate. It has been proved that the substitution of Cys215 or Asp181 can lead to loss of PTP1B catalytic activity but does not affect the substrate binding affinity. Other amino acid residues including Arg47, Lys120 and Val49 are also involved in the binding of substrate. The C terminal domain, which consists of 35 specific amino acid residues, locates the PTP1B to the endoplasmic reticulum [11–14].

PTP1B substrates include receptor tyrosine kinases (RTKs), intracellular PTKs, adapter proteins, cytoskeletal proteins, and transcription factors, which are involved in multiple cellular processes such as glucose uptake, proliferation, differentiation, apoptosis, cell–cell adhesion, extracellular matrix attachment, motility and invasion. Tyrosine dephosphorylation mediated by PTP1B may either inactivate or activate its substrate and correspondingly modulate their downstream signaling. For instance, PTP1B-mediated dephosphorylation of Src at Y527 enables subsequent activation of small GTPases such as Ras and Rac. Bjorge et al. have shown that PTP1B is the major PTP responsible for regulation of Src in MDA-MB-435s cancer cells[15]. Changes in expression and activity of PTP1B have been shown to be associated with various human cancers[16].PTP1B may act either as a tumor suppressor or a tumor promoter through the dephosphorylation of specific substrates[17]. For these reasons, PTP1B has attracted attention as a potential therapeutic target in obesity, diabetes, and cancer. Like the analysis of genetic alterations in PTK, the present study suggests the possibility of individualized therapy based on the mutant phosphatases present in specific tumors.

In order to test the hypothesis that targeting PTP1B may offer a novel therapeutic approach in tumors, we sequenced PTP1B cDNAs from 43 human colon tumors and 47 thyroid tumors and found incorrectly spliced mutants. One mutant, which was found with high frequency in both tumor types, was studied in detail and shown to transform in rat embryo fibroblasts (REF), and to form xenograft tumors in nude mice, but it was not required Src-mediated activation.

## Materials and Methods

### Reagents

Anti-Src mAb327 monoclonal antibody has been described [18]. Anti-PTP1B (H-135), anti-HA tag rabbit polyclonal antibodies were from Santa Cruz Biotechnology (Santa Cruz, CA), anti-pY527 polyclonal antibody, anti-IGF-1Rβ polyclonal antibody, anti-phospho IGF-1Rβ (Tyr1135) monoclonal antibody were from Cell Signaling Biotech (Cell Signaling, Boston). Anti-PTP1B (610139) mouse monoclonal antibody was from BD Bioscience. HRP-linked secondary antibodies for immunoblots were from Jackson ImmunoResearch (West Grove, PA). GammaBind sepharose (used with Src monoclonal antibody) was from Amersham Biosciences (Piscataway, NJ).

### Tumor samples collection

Patients who were diagnosed with thyroid or colorectal tumors by standard clinical testing and criteria underwent tumor resection. The collection of the tumor tissue samples was performed in accordance with the protocols approved by the Ethics Committee of Shanghai Jiao Tong University School of Medicine, and the written informed consent was obtained from every patient. Tumor tissue samples were collected at surgery and frozen in liquid nitrogen immediately for later use. Normal tissues surrounding tumors were collected simultaneously as control.

### RNA isolation and RT- PCR

Total RNA from thyroid or colorectal tumor tissues and normal tissues from distant parts of the excised tissue were stored in liquid nitrogen. 50–100 milligrams of tumor or normal tissue were homogenized twice 20 s with a Power Gen Model125 homogenizer (Fisher Scientific, USA) on ice in 1.5ml conical microcentrifuge tube containing 0.5ml TRIzol RNA isolated reagent according to the manufacturer’s instructions (BD Pharmingen). First strand cDNA was generated from 5ug of total RNA using reverse transcriptase Supercript II (Invitrogen) and oligonucleotide (dT) primers. The cDNAs were amplified with high fidelity Taq polymerase. Specific primers were designed to amplify a 1308 bp PTP1B product which covering the entire open reading frame (sense 5’-ATGGAGATGGAAAAGGAGTTCGAGCAGATC-3’; antisense 5’-CTATGTGTTGCT GTTGAACAGGAACCTGTAG-3’). PCR was performed in 50ul of a solution containing 10×PCR buffer 5ul, 10mM dNTP 1ul, 10umol/L primers 1ul, 50mM MgCl_2_ 2ul.The reaction consisted of 25 cycles of denaturation of 95°C for 40s, annealing at 55°C for 40s and extension at 68°C for 2 minutes. PCR products were subjected to pCR2.1TOPO-TA ligation system (Invitrogen). Positive clones were then sequenced by the primers directed at sequences in the pCR2.1 TOPO vector. Gene mutations were analyzed using Vector NTI software subsequently.

### Construction of Plasmids

DNA fragment encoding a HA epitope tag at the 3’ end of wild type (wt) PTP1B and the truncated mutant PTP1B (ΔE5 and ΔE6) were generated by PCR with Taq polymerase (Invitrogen, USA). HA tagged wt PTP1B, ΔE5 and ΔE6 were amplified from plasmid pCR2.1wt PTP1B, pCR2.1ΔE5 and pCR2.1ΔE6 using the 5’ primer (5’ primer1) 5’GCACGTCGACCACC**ATG**GAGATGGAAAAGGAGTTCGAG-3’ and the 3’ primer (3’ primer1) 5’-CAGGGTCGAC**CTA**TGCGTAGTCTGGCACGTCGTATGGGTATGTGTTGCT GTTGAACAGGAACCTG-3’. The 5’ primer contains a Sal I site (underlined) and a start codon (bold). The 3’ primer contains the coding sequences (italics) for the HA epitope YPYDVPDYA (Wilson et al,. 1987), the stop codon (bold) and a Sal I site (underlined). Sal I -digested PCR product was ligated into pTet-Splice (Life technologies) to make plasmids pTPTP1B-HA (wt), pTPTP1BΔE5-HA and pTPTP1BΔE6-HA. Plasmid co-transfection with the transactivator pTet-tTAK (Life technologies) was used to express protein only in the absence of doxycycline (Shockett et al., 1995).

### Transfection and stable cell lines

Cell lines that inducible overexpress HA-tagged human wt PTP1B, ΔE5 or ΔE6 were generated by co-transfecting plasmids pTPTP1B-HA (wt), pTPTP1BΔE5-HA, pTPTP1BΔE6-HA or pTet-Splice into rat embryo fibroblasts with pTet-tTAK and the selected plasmid pSV2neo. Transfection procedure has been described previously[18]. G418-resistant colonies were screened by immunoblotting with both anti-PTP1B and anti-HA antibodies for inducible expression of wt PTP1B, ΔE5 and ΔE6. All lines were reselected and verified by immunoblot with anti-PTP1B and anti-HA polyclonal antibodies in the presence or absence of doxycycline. Cells were grown in monolayer culture in DMEM plus 10% calf serum and 5 ng/ml doxycycline.

### Analysis of ΔE5 and ΔE6 expression

ΔE5 or ΔE6 induced cells were washed with DMEM, treated by trypsin, plated into DMEM without doxycycline, and grown for 16–18 h (80–90% confluence) before harvesting. Uninduced cells were treated in the same way except that the medium contains doxycycline. Cells were washed twice with ice-cold PBS and lysed in HEPES buffer essentially as described [18].

Cell lysates (20ug of protein) or immunoprecipitates were resolved by 10% SDS-PAGE. Proteins were transferred to PVDF membrane (Milllipore) and blocked with either PBST (PBS, 0.05% Tween 20) containing 3% non-fat milk for polyclonal and TBST (Tris-buffered saline, 0.05% Tween 20) containing 3% bovine serum albumin. Membranes were incubated with primary antibodies for overnight at 4 ^0^C at the following dilutions: anti-PTP1B polyclonal antibody (1:1000), anti-HA tag polyclonal antibody (1:1000), anti-Src mAb 327 (1:6000), anti-pTyr antibody (pY100, 1:2000), anti-IGF-1Rβ polyclonal antibody (1:1000), anti-phospho IGF-1Rβ (Tyr1135) monoclonal antibody (1:1000), then secondary antibodies either anti-rabbit or anti-mouse IgG at 1:10000. Proteins were visualized by chemiluminescence (NEN life Science Products).

### Biological assays

Cells were assayed for focus formation by mixing 300 cells of each type to be tested with 5 x 10^5^ normal REF cells, plating into 100-mm culture dishes in DMEM lacking doxycycline, counting for focus formation after 20 days (Johnson et al., 1985). Colony formation assay in 0.3% soft agarose in the absence of doxycycline has been described previously[18]. Animal studies have been described in detail previous [9]. Briefly, All cell lines (2.5 × 10^6^, suspended in 100 μl sterile PBS) were harvested and injected subcutaneously into 5-week-old female BALB/c nude mice (n = 4 or 5) individually. All tumors appeared by 7–8 days after injection and were monitored until 27-day when the tumors grew to ~ 1cm diameter. All mice were euthanized by cervical dislocation at 27-day after injection and the tumors were measured. Tumor volumes were determined by measuring the length (a) and the width (b). The tumor volume (V) was calculated according to the formula V = a/2*b^2^. BALB/c ASlac-nu mice were from The Shanghai Slac Laboratory Animal Center. Animal procedures were carried out according to a protocol approved by the Institutional Animal Care and Use Committee (IACUC) of Shanghai Jiao Tong University School of Medicine. Mice were housed in ventilated cages, maximum 5 per cage, under a 12-hour/12-hour light/dark cycle and an ambient temperature of 22–25°C. Researchers ensured that each animal was provided with adequate food, water and clean enclosures. Animals were monitored daily with health monitor forms. None of the animals involved in this study showed sign of illness or died prior to the experimental endpoint. We did not perform surgical procedures as part of this study.

### Phosphatase assay

PTP1B WT-HA or ΔE5-HA or ΔE6-HA immunoprecipitates with HA or PTP1B (610139) antibodies were washed three times with lysis buffer and once with phosphatase buffer (50mM immidazole pH7.2, 5mM DTT), then resuspended in150 ul of phosphatase buffer. Ten micromilitres of the immunoprecipitate was incubated in a total volume of 25ul of phosphatase buffer containing 6ug of tyrosine phosphorylated MBP as described[18].

### Src kinase assay

Src kinase assay were carried out exactly as described previously[18]. Lysates containing 500ug of total protein were incubated with anti-Src mAb327 for 2 h at 4°C, following GammaBind Sepharose beads were added for an additional 1 h with mixing. Immunoprecipites were washed once with RIPA buffer containing to 0.5M NaCl, and twice with lysis RIPA buffer.

## Results

### Identification of six PTP1B truncation mutants in human tumors

**Fig 1:**
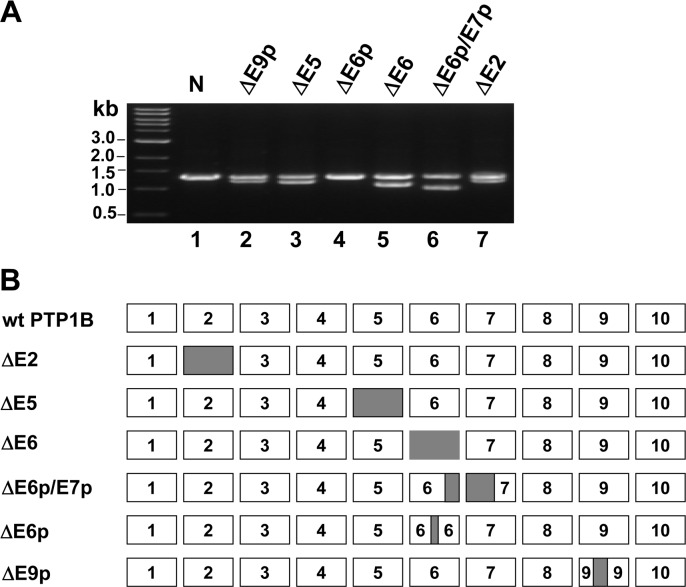
Identification of mutation of PTP1B from human specimens. **A**. Agarose gel showing the PCR amplification of the PTP1B RNA transcript using RT-PCR with cDNA as template, PCR of full length (N) or deletions of PTP1B (Δ). The positions of molecular weight standards are indicated (in Kb). **B**. Schematic representation of the identified mutations of PTP1B. Open bars represent the exons and connected lines are regions of exons that have been deleted, such as deletion of parts of or whole exons.

RT-PCR was used to amplify and sequence the coding region of PTP1B cDNAs from 43 human colon tumors and 47 thyroid tumors from randomly selected patients of Chinese descent. Paired normal samples from surrounding tissue were tested simultaneously as control. Six different types of PTP1B mutants were found in colon tumors and three types of them were also found in thyroid tumors. The characteristics of these six PTP1B mutants were summarized in Table 1. The observed frequency of occurrence of mutants is varied from 2%-21%. Mutant ΔE6, with the deletion of the whole exon6, was the PTP1B mutant with the highest frequency in both types of tumors (from 9/43 colon tumors and 7/47 thyroid tumors). Mutant ΔE5, with the deletion of the whole exon5, was also found in 8/43 colon tumors.The length of mutant ΔE6 and ΔE5 mRNA are 1095bp and 1167bp respectively and neither have a reading frame shift. The other four types of PTP1B mutants, which lack the entire exon2, partial exon6, partial exon6 and exon7, partial exon9, are designated PTP1B ΔE2, PTP1BΔE6p, PTP1BΔE6p/E7p and PTP1BΔE9p respectively(Fig 1B). The occurrence of these six mutants did not correlate with age or gender of patients or cancer state and site (Table 2).

**Table 1:** PTP1B mutations in human colon and thyroid tumors.

Mutant	Occurrence frequency	mRNA change	del site	size (nt)	deduced Amino Acid	ORF shift
ΔE2	3/43 (7%) colon	91 bp del	64–154	1217	362	no
ΔE5	8/43 (19%) colon	138 bp del	355–492	1170	389	no
ΔE6	9/43 (21%) colon	210 bp del	493–702	1098	365	no
ΔE6p	1/43 (2.5%) colon	6 bp del	604–609	1305	433	no
ΔE9p	1/43 (2.5%) colon	104 bp del	1107–1210	1204	401	yes
ΔE6p/E7p	1/43 (2.5%) colon	224 bp del	556–779	1081	185	yes
ΔE2	2/47 (4%) thyroid	91 bp del	64–154	1217	362	no
ΔE6	7/47 (15%) thyroid	210 bp del	493–702	1098	365	no
ΔE6p/E7p	1/47 (2%) thyroid	224 bp del	556–779	1084	185	yes

**Table 2:** PTP1B mutation in patients with colon and thyroid tumors.

patient(n)	Gender/Age/Stage	Site	Alleles
8	F/68/II	rectum	wt/ΔE9p
10	M/76/III	sigmoid	wt/ΔE6
13	M/80/II	ascending colon	wt/ΔE6p
34	M/51/III	rectum	wt/ΔE6
35	M/52/I	sigmoid	wt/ΔE5
36	M/74/II	rectum	wt/ΔE5
45	M/65/III	recto-sigmoid	wt/ΔE2
46	M/73/II	sigmoid	wt/ΔE2
48	F/57/II	rectum	wt/ΔE6
53	M/69/III	recto-sigmoid	wt/ΔE6
54	M/57/II	recto-sigmoid	wt/ΔE5
55	M/50/II	rectum	wt/ΔE5
64	M/59/I	rectum	wt/ΔE6
65	M/72/III	ascending colon	wt/ΔE5
66	F/58/II	rectum	wt/ΔE6
68	F/84/III	rectum	wt/ΔE2
69	M/71/II	rectum	wt/ΔE6
73	F/74/III	ascending colon	wt/ΔE5
93	F/83/II	sigmoid	wt/ΔE6p/ΔE7p
94	M/69/IV	rectum	wt/ΔE6
95	F/61/I	rectum	wt/ΔE5
96	M/68/II	ascending colon	wt/ΔE6
97	M/76/II	ascending colon	wt/ΔE5
62	M/28/II	papillary thyroid	wt/ΔE6
100	F/69/II	papillary thyroid	wt/ΔE6
138	F/33/II	papillary thyroid	wt/ΔE6
171	F/42/III	papillary thyroid	wt/ΔE6
184	F/27/II	follicular thyroid	wt/ΔE6
186	F/38/II	follicular thyroid	wt/ΔE6p/ΔE7p
234	F/46/II	papillary thyroid	wt/ΔE2
247	F/49/III	papillary thyroid	wt/ΔE6
256	M/37/II	papillary thyroid	wt/ΔE2
330	M/53/II	papillary thyroid	wt/ΔE6

All mutant-containing RT-PCR products contained similar amounts of wide type and mutant amplicons (Fig 1A), indicating, since the PCRs were competitive, that mutant mRNAs were present in significant amounts. It is more likely that both alleles are transcribed with only one allele mutated in tumor tissues. Noticeably, one more accurate method, DNA sequencing of those tumors, would be very informative to determine if these six mutants are the consequence of alternative splicing, DNA deletion or splice site mutation.

### Expression of ΔE6 and ΔE5 in REF and their tyrosine dephosphorylation activity

**Fig 2:**
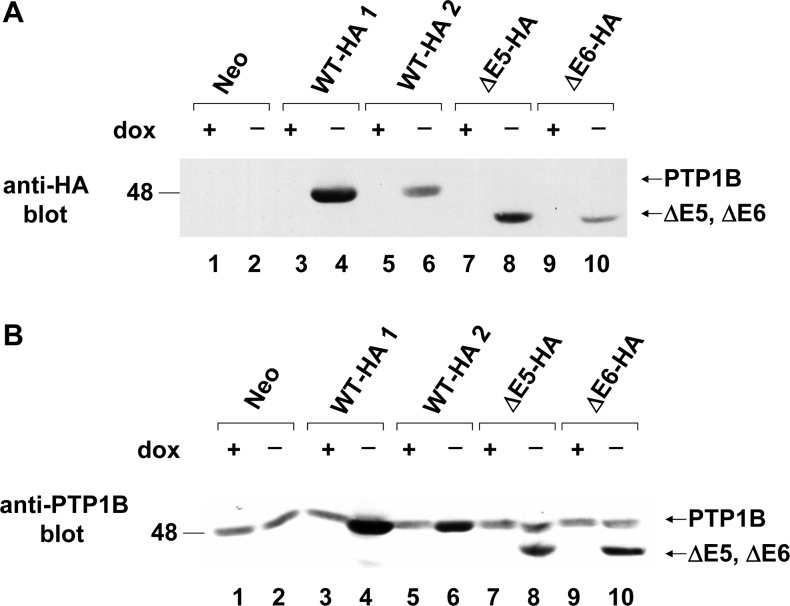
PTP1B inducible expression. Cell line names are explained in Table 3. **A.** 30ug of each lysate was prepared from uninduced and induced Neo (lanes 1 and 2), WT-HA1(lanes 3 and 4), WT-HA2 (lanes 5 and 6), ΔE5-HA (lanes 7 and 8) and ΔE6-HA (lanes 9 and 10) overexpressor cells. Lysates were immunoblotted with anti-HA polyclonal antibody. **B.** As in A, except that lysates were immunoblotted with anti-PTP1B polyclonal antibody. The positions of molecular weight markers (in kDa) are indicated.

Because the high occurrence rate, mutant ΔE6 and ΔE5 were selected for further study. Coding sequence of mutant ΔE6 and ΔE5 were cloned into a Tet-off inducible expression vector pTetsplice and transfected into REF cells. Multiple inducible cell lines of two mutants were cloned. REF overexpressing the two PTP1B variants (ΔE6-HA and ΔE5-HA) under Tet-off control were incubated either in the presence or absence of doxycycline(dox). The cell lysates were immunoblotted with the anti HA or anti-PTP1B antibodies. Lines expressing HA-tagged human wide type PTP1B (wt-HA1 and wt-HA2) or no exogenous PTP1B (neo) were used as positive and negative controls. Two PTP1B mutants were inducibly expressed in the absence of dox. Compared with the wide type PTP1B control, immunoblots shown that the electrophoretic bands of two PTP1B mutants located in the lower molecular weight gel region (Fig 2A and Fig 2B).

**Fig 3:**
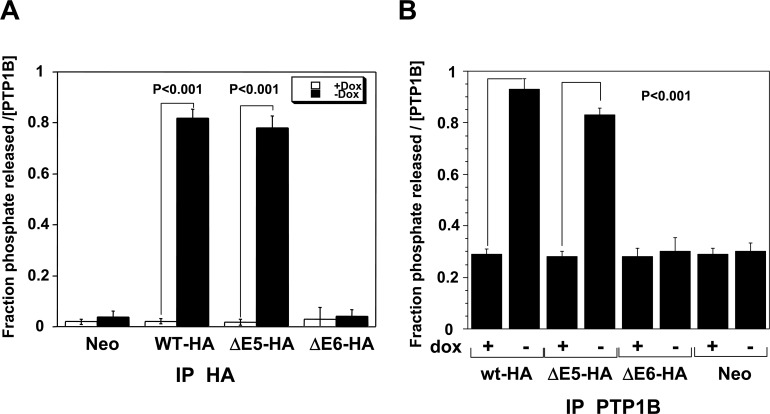
Phosphatase activity of PTP1B isoforms. *In vitro* dephosphorylation of no-specific substrate MBP by Neo, WT-HA1, ΔE5-HA and ΔE6-HA. MBP were tyrosine phosphorylated in vitro using v-Src and [γ-^32^P] ATP. The phosphorylated substrate (MBP; 1.2 X 10^5^ c.p.m/lane, 6ug/lane) was incubated in phosphatase buffer for 10 min at 30°C with anti-HA (Fig 3A) or anti-PTP1B (Fig 3B) immunoprecipitates from 500ug of total cell lysates prepared from Neo (control), WT-HA, ΔE5-HA and ΔE6-HA overexpressing cells (induced removal of doxycycline for 18 h or uninduced with the addition of 5ng/ml doxycycline). Dephosphorylation reactions were stopped and the amount of [^32^P] phosphate released was measured using scintillation counting. Results are the averages of three independent experiments (error bars indicate the SEMs).

Because exon6 has been reported to form the active center of PTP1B [12,13], the protein product of mutant ΔE6 is predicted to be inactive. The results of phosphatase activity assay verified that the protein product of ΔE6-HA has no enzymatic activity while that of ΔE5-HA has similar activity as WT-HA1 (Fig 3A and 3B, S3 Table).

### Transformation of REF and *in vivo* tumorigenicity by ΔE6 and ΔE5

**Fig 4:**
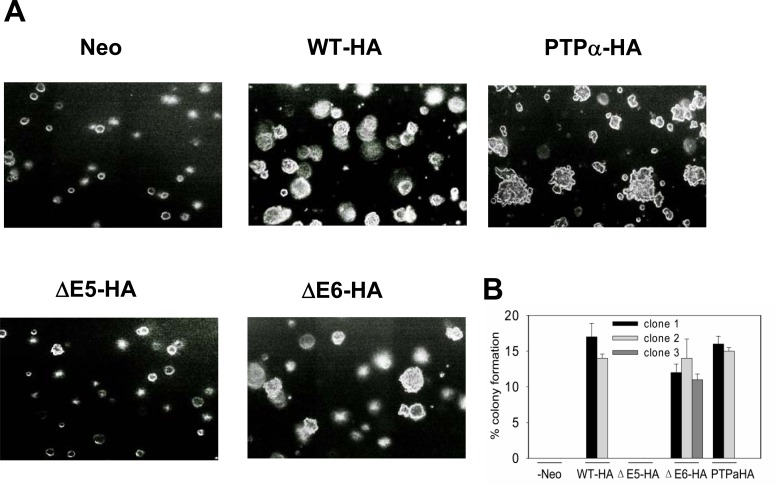
Effect of ΔE6-HA on colony formation. A. Colony formation in soft agarose by WTPTP1B-HA, ΔE5-HA and ΔE6-HA overexpressing cells. Neo (control), WT-HA, ΔE5-HA and ΔE6-HA were suspended in semisolid media containing 0.3% agarose without doxycycline. A total of 500 cells was added to each 6-cm culture dish and colonies were counted after 3 weeks. PTPα overexpressing cell lines were used as positive control. B. One quantification graph with an average of three independent experiments for all 11 cell lines (error bars indicate the SEMs).

Expression of WT-PTPα has been shown to induce focus formation in monolayer culture, anchorage independent growth, and tumors in nude mice as described in detail previously[7–9]. The transformation activities of PTP1B were determined using the same assays. PTPα overexpressing cell lines were used as positive control. REF cells expressing either WT-HA, ΔE6-HA and PTPα had similar focus forming efficiencies when mixed with normal REF cells; these ranged from 10–20%. Concordantly, all formed colonies in soft agarose with similar efficiencies ranging from 11%-17%. All cell lines were also tested for *in vivo* tumorigenicity by subcutaneous injection into nude mice. Similar to the results of soft agarose and foci formation tests, both WT-HA and ΔE6-HA overexpressing cell lines were tumorigenic and formed tumors that rapidly grew at the same rate as those induced by PTPα. ΔE5-HA mutant showed the lack of transformation activity in all tests (Fig 4 and Table 3, S1 Table, S2 Table). It should be noted that wild type is present, as its expression is reduced in the ÄE5-HA and the ÄE6-HA cell lines, effects seen are more likely to originate from the mutant PTP1B. These observations indicate that the phosphatase activity of ΔE6 and ΔE5 is not required for transformation.

**Table 3:** Transforming activities of PTP1B mutations overexpression cells.

Protein	Cell line	% colony formation	% focus formation)	Tumors in nude	Tumors volume
		in soft agarose(n = 3)	in cell mixing (n = 3)	mice (latency)	(mean + SD) /cm^3^
Neo	REF(pTet-SPLICE)1	0	0	0/5	0
	REF(pTet-SPLICE)2	0	0	0/5	0
wt-HA	REF(pTPTP1BHA)1	17 ± 1.9	16 ± 2.9	5/5 (7-8d)	0.2271±0.0544
	REF(pTPTP1BHA)2	14 ± 0.6	20 ± 1.9	5/5 (7-8d)	0.2024±0.0485
ΔE5-HA	REF(pTPTP1BΔE5HA)1	0	0	0/5	0
	REF(pTPTP1BΔE5HA)2	0	0	0/5	0
ΔE6-HA	REF(pTPTP1BΔE6HA)1	12 ± 1.2	10 ± 1.2	5/5 (7-8d)	0.2270±0.0778
	REF(pTPTP1BΔE6HA)2	14 ± 2.7	12 ± 1.5	4/4 (7-8d)	0.2545±0.1018
	REF(pTPTP1BΔE6HA)3	11 ± 0.8	14 ± 1.8	4/4 (7-8d)	0.2115±0.0682
PTPáHA	REF(pTPTPαHA)1	16 ± 1.1	17 ± 2.2	5/5 (7-8d)	0.2872±0.0933
	REF(pTPTPαHA)2	15 ± 0.5	16 ± 2.0	4/4 (7-8d)	0.3191±0.0734

### Inducing of Src dephosphorylation and activation by PTP1B mutants

**Fig 5:**
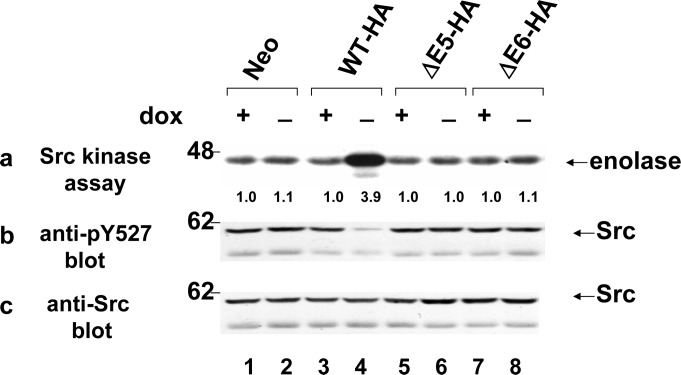
Src kinase activity and Tyr527 dephosphorylation. Src has been immunoprecipitated from WT-HA, ΔE5-HA and ΔE6-HA overexpressing cells, and Neo (control). Cells were grown in the present (+) or absence (-) of 5ng/ml doxycycline (Dox) for 18 h. Each Src immunoprecipitate was divided into three portions which were immunoblotted (45%) with either anti-pY527 polyclonal antibody (panel b) or (45%) anti-Src monoclonal antibody (panel c), 10% of each immunoprecipitate was subjected to Src kinase assay (panel a) in kinase buffer containing [γ-^32^]ATP and enolase substrate followed by SDS-PAGE and autoradiography of the phosphorylation products. Overexpression of HA-tagged WT-HA decreased tyrosine phosphorylation of Src (pY527) by 59±6% (panel b, lane 4) and increased Src kinase activity by 3.7±2.1 fold (panel a, lane 4). The positions of molecular weight standards (in kDa) are indicated.

Anti-phosphotyrosine (pY527) and anti-Src immunoblots of anti-Src immunoprecipitates revealed that overexpression of HA-tagged WT-HA1 decreased Src tyrorine527 phosphorylation by 59±6% (Fig 5, panel b, lane 4) and increased Src kinase activity by 3.7±2.1 fold (Fig 5, panel a, lane 4). Interestingly, expressing of the ΔE5-HA mutant that has phosphatase activity, did not result in Src activation through dephosphorylation of the inhibitory Y527 residue.

### Inducing of IGF-1Rβ dephosphorylation by PTP1B mutants

Anti-phosphotyrosine (p-IGF-1Rβ Y1135) and anti-IGF-1Rβ immunoblots of anti-IGF-1Rβ immunoprecipitates were completed. Consistent with the hypothesis that IGF-1Rβ is a direct substrate of PTP1B, dephosphorylation of IGF-1Rβ at tyr-1135 were observed in all the three cell lines.

## Discussion

Using RT-PCR and sequencing the coding region, we identified six mutants in PTP1B transcripts from Chinese patients with colon tumors and thyroid tumors. Especially, tumors are heterogeneous and are infiltrated by immune cells including lymphocytes, macrophages, etc, which typically are not present in a normal cellular context. It couldn’t rule out the possibility that the PTP1B variants identified here might not be present in the cancer cells but other type of cells. The details of the mutants are summarized in Table 2. Three of the mutants, named PTP1BΔE6p, PTP1BΔE6p/E7p and PTP1BΔE9p, were found only once in 43 colon or 47 thyroid tumors respectively and not selected for further study because of their very low frequency. Interestingly, PTP1BΔE2, found in 3/43 colon tumors and 2/47 thyroid tumors, has been confirmed from a large scale cDNA sequencing project and termed PTP1B transcript variant 2 in pubmed[19]. This isoform 2 encodes 362 AAs using a downstream start codon and has a shorter N-terminal compared to the common (normal) variant. In other words, this mutant is a known isoform and was not selected for further study.

Two mutants, ΔE5 and ΔE6, were selected for further functional study because of their high occurrence rate in our tumor samples. PTP1B was the first PTP to be cloned and sequenced and its crystal structure and enzymatic mechanism have been well delineated. The active site of PTP1B is defined by residues 214–221 coded by Exon6[14], thus ΔE5 is predicted to have phosphatase activity. In fact, we observed that the ΔE5 mutant has similar dephosphorylation activity as compared to WT-PTP1B (Fig 3A and 3B, S3 Table). However, two subcloned cell lines of this mutant displayed no transforming phenotype (Fig 4A) or tumorigenicity *in vivo* (Table 3, S2 Table). PTP1B was reported to promote growth and invasion of cancer cells in a Src-dependent manner. In colon and breast cancer cells, the overexpression of PTP1B is associated with decreased inhibitory Y527 phosphorylation of Src, and this results in accelerated colony formation in soft agar as well as increased tumor growth in immunodeficient mice[3,20]. Accordingly, we checked the status of Src Tyr527 phosphorylation and found it was unchanged in ΔE5 overexpressing cell line (Fig 5). The mechanism accounting for failure of the phosphatase-competent ΔE5 mutant to activate Src remains unknown. The crystal structure of a 37 KD form (residues 1 to 321) of PTP1B has been determined by Barford and Tonks [14]. Their work suggests that residues Asp48, Lys 116, Lys120 and Tyr46 are essential to form a substrate binding site. It is noted that Exon5 encodes residues from 119 to 164 so that the ΔE5 mutant lacks Lys120, implying that this mutant may be somewhat defective in binding specific substrates such as Src. However, recently, Fan et al. reported that PTP1B potentiated Src activity indirectly via dephosphorylation of CBP/PAG with concomitant disruption of its association with CSK [21]. The mechanism by which PTP1B activates Src is more complicated than is currently believed, and more studies with PTP1B and the ΔE5 PTP1B mutant isoform are needed to clarify the relationship between PTP1B and Src activation. Finally, it is striking that the frequency of the ΔE5PTP1B mutant isoform is high 19% (8/43) in colon tumors but was absent (0/47) in thyroid tumors. This raises the possibility that this mutant contributes to tumorigenesis in colon cancer.

**Fig 6:**
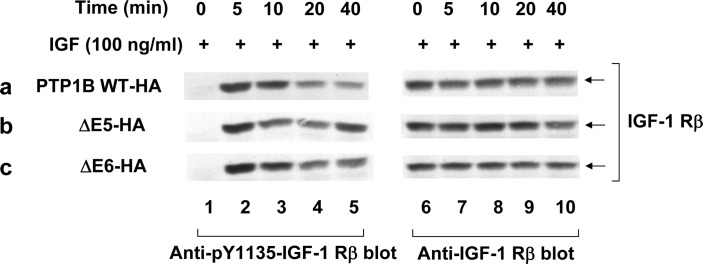
IGF-1 receptor signaling. All the three cell lines were FBS-starvation and stimulated with IGF (100 ng/ml) for the indicated times, RIPA-lysed, immunoprecipitated and immunoblotted with the designated antibodies.

The phosphatase-deficien mutant, ΔE6, was also observed with high frequency in both colon and thyroid tumors, unexpectedly, although the ΔE6 mutant was incapable of dephosphorylating p-Tyr527 of Src (Fig 5), ΔE6 overexpressing cell lines were tumorigenic and formed tumors that rapidly grew at the same rate as those induced by PTP1B WT and PTPα, which can activate Src directly as we have previously reported [9,18]. Multiple independent experiments confirmed that the oncogenic properties of this mutant are independent of its phosphatase activity. It has been demonstrated that substitution of Cys215 to Serine the catalytic site of PTP1B is sufficient to abolish its catalytic activity with downstream biological effects [22]. It is notable that there are similar phosphatase activities but different biological function between PTP1B C215S and the ΔE6 deletion mutant comprising residues 165 to 234 including C215. One possibility is that the conformational change in ΔE6 contributes to its interaction with other molecules, which results in tumourigenesis. We have previously reported on an analogous phenomenon. In that study, RPTPα245, a truncated isoform of RPTPα that is devoid of phosphatase activity, can bind to endogenous RPTPα directly, which decreases RPTPα-Grb2 binding and increases RPTPα activation of Src[9]. Conversely, the exact role of PTP1B in tumourigenesis is remains uncertain. Mechanistically, PTP1B has many substrates other than Src. It is known that PTP1B is a negative regulator of IGF-1 receptor signaling as a tumor suppressor[23]. We also checked the IGF-1 receptor signaling in the PTP1B WT-HA, ΔE5-HA and ΔE6-HA cell lines. The primary result was shown in Fig 6. We observed attenuated IGF-induced activation of IGF-1Rβ in all the three cell lines, coincident with time-dependent decreased phosphorylation of IGF-1Rβ at tyr-1135. Although there was slight higher level of IGF-1Rβ phosphorylation at 40 min after IGF stimulation in ΔE5-HA and ΔE6-HA cell lines compared with WT-HA (lane 5), it is unlikely the reason that ΔE6 cell line were tumorigenic. Generally, PTP1B may act as a tumor suppressor, a tumor promoter, or both depending on the type of tissues involved and/or the presence mutations in other cancer susceptibility genes[17]. In the current study we clearly demonstrate that both PTP1B WT and ΔE6 when expressed in rat embryo fibroblasts have tumor promoting activity. This carcinogenic effect may not generalize to other cancer cell types or tissues. Future experiments are needed to address this question. Moreover, follow-up time of the patients relevant to ΔE5 and ΔE6 function is a considerable question. In our study, most tumor tissue samples are from late-stage colon or thyroid cancer patients (stage II to IV), so it is likely that ÄE5 and ÄE6 imply the outcome than cause of cancer. It is necessary to analyse the presence of ÄE5 and ÄE6 in another cohort with a longer follow-up time in future study, which may help to illuminate the overall outcome of the mutants.

Despite the increasing amount on PTP1B function research in cancer, abnormal PTP1B is somewhat reported. The first variant of PTP1B was reported from B. Neel[4].They found that in human diploid fibroblasts (HF), an alternative pre-mRNA splicing isoform is produced upon stimulation with growth factors, which encodes a PTP1B protein with an altered C terminus because of retention of the last intron. Recently, Gunawardana et al reported [16] a high frequency of somatic coding-sequence mutations in PTPN1, the gene for encoding PTP1B, in primary mediastinal B cell lymphoma (PMBCL) and Hodgkin lymphoma, and concluded that these PTPN1 mutations are drivers of lymphomagenesis. Notably, it was shown recently that using cbioportal, Hoekstra et al analyzed the TCGA colorectal cancer dataset, revealing an alteration of PTPN1 in 45% (88 out of 195) of the CRC cases with frequent amplifications and only few mutation[24]. However, in our study, nearly 50% of colon tumors have a mutation in PTPN1. The probable reason for this difference is all tumors in our study are only from Chinese patients, so it is important to screen non-Chinese patients in the same way to test for potential different genetic background. Another minor reason may be from different techniques used. RT-PCR method is simple and effective in our study, but a large-scale DNA sequencing and diverse mRNA detection are adopted in TCGA dataset. Chen et al also demonstrated increased PTP1B expression and activity in primary colorectal cancer tissue and suggested that PTP1B expression may serve as a valuable prognostic biomarker for CRC[25]. Compared with their published data, our six mutants found in Chinese colon and thyroid tumors are novel. Taken together, these observations indicate that there are different mutational patterns of PTP1B in different tumor types.

In summary, PTP1BΔE6 mutant possesses no phosphatase activity yet is able to induce cellular transformation in rat fibroblasts. This is independent of Src activation through Y527 dephosphorylation and likely is explained by interaction of PTP1B with other molecule(s) in a tumorigenic pathway. Although the PTP1BΔE5 mutant possesses non specific dephosphorylating activity through MBP, it was unable to effects c-Src activation in vivo that correlated with lack of transforming ability in rat fibroblasts. Our data raise the possibility that the PTP1BΔE6 mutant may be a viable therapeutic target in colon and thyroid cancer.

## Supporting Information

S1 Table Original data for Colony formation and Focus formation in Table 3 and Fig 4B. (XLS)

S2 Table Original data for tumor volume in Table 3. (XLS)

S3 Table Original data for PTP1B phosphatase assay in Fig 3A and 3B. (XLS)
